# Comparability of family planning quality of care measurement tools in low-and-middle income country settings: a systematic review

**DOI:** 10.1186/s12978-021-01261-1

**Published:** 2021-10-30

**Authors:** Elizabeth Hazel, Diwakar Mohan, Margaret Gross, Sushama Kattinakere Sreedhara, Prakriti Shrestha, Maia Johnstone, Melissa Marx

**Affiliations:** 1 Institute for International Programs, Johns Hopkins Bloomberg School of Public Health, 615 N Wolfe St, Baltimore, MD 21205 USA; 2 Formerly of Johns Hopkins Bloomberg School of Public Health, Baltimore, MD USA; 3 North Carolina State University, Raleigh, NC USA

**Keywords:** Family planning, Quality of care, Assessment tools, Validity, Low-and-middle income countries

## Abstract

**Background:**

In low-and-middle income countries (LMICs), accurate measures of the elements of quality care provided by a health worker through family planning services (also known as process quality) are required to ensure family’s contraceptives needs are being met. There are many tools used to assess family planning process quality of care (QoC) but no one standardized method. Those measuring QoC in LMICs should select an appropriate tool based the program context and financial/logistical parameters, but they require data on how well each tool measures routine clinical care. We aim to synthesize the literature on validity/comparability of family planning process QoC measurement tools through a quantitative systematic review with no meta-analysis.

**Methods:**

We searched six literature databases for studies that compared quality measurements from different tools using quantitative statistics such as sensitivity/specificity, kappa statistic or absolute difference. We extracted the comparative measure along with other relevant study information, organized by quality indicator domain (e.g. counseling and privacy), and then classified the measure by low, medium, and high agreement.

**Results:**

We screened 8172 articles and identified eight for analysis. Studies comparing quality measurements from simulated clients, direct observation, client exit interview, provider knowledge quizzes, and medical record review were included. These eight studies were heterogenous in their methods and the measurements compared. There was insufficient data to estimate overall summary measures of validity for the tools. Client exit interviews compared to direct observation or simulated client protocols had the most data and they were a poor proxy of the actual quality care received for many measurements.

**Conclusion:**

To measure QoC consistently and accurately in LMICs, standardized tools and measures are needed along with an established method of combining them for a comprehensive picture of quality care. Data on how different tools proxy quality client care will inform these guidelines. Despite the small number of studies found during the review, we described important differences on how tools measure quality of care.

**Supplementary Information:**

The online version contains supplementary material available at 10.1186/s12978-021-01261-1.

## Plain language summary

Accurate measures of process quality of care—or how well clinicians deliver services according to standards of care—are important to monitor, evaluate and improve service quality. Periodic surveys of health facilities or provider are the main source of national or regional quality of care data in many low- and middle-income countries. Many tools are used for these surveys: exit interviews with patients, observations of the visits by a clinician-assessor, simulated or mystery patients, and others. Implementers must select the appropriate and feasible tools for their program, context and setting but there is little information on how well different tools measure the same quality of care indicators.

This review summarizes the current literature on the validity of measurements from different family planning quality of care tools. We found only eight studies, but we were able to see some differences important to consider when selecting the most appropriate tool. For instance, patients reported different events through an exit interview than what was documented by the assessor during the same visit. Exit interviews may be more appropriate to measure client experience or satisfaction rather than specifics of the care received. Knowing these differences will help implementers choose an appropriate tool depending on the focus of the quality assessment. This review contributes to the body of knowledge on improving quality of care measurements, resulting in better data to improve family planning services for patients.

**Supplementary Information:**

The online version contains supplementary material available at 10.1186/s12978-021-01261-1.

## Background

Globally, there is recognition that quality care is a human right and improving quality services is critical to obtaining universal health coverage and helping countries meet the 2030 Sustainable Development Goals (SDG) [1–3]. Tremendous progress in family planning has been made in the last 50 years, but the unmet need for contraceptives remains unacceptably high especially in low-and-middle income countries (LMICs) [4]. A time trend analysis of data in over 70 LMICs found annual increases in proportion of women with demand satisfied and reductions in inequalities by wealth and geographic area [5]. However, only 44 of 62 LMICs included in the analysis were projected to meet the SDG of greater than 75% demand for family planning services satisfied by modern methods [5]. This coverage gap varies by region: West and Central Africa have the lowest percent of demand satisfied (33%) while South Asia, Latin America, and the Caribbean have the highest percentage of demand satisfied (70%) [6]. Improving the quality of service provision may help close this gap.

The World Health Organization (WHO) and the Institute of Medicine (IOM) have outlined domains of health service quality that include safety, effectiveness, timeliness, efficiency, equity, accessibility, and patient-centeredness [7, 8]. Judith Bruce’s 1990 seminal family planning quality of care framework includes elements of service such as method choice, services appropriateness, and continuity of care. This and other frameworks define technical competence (i.e., provider knowledge and skills) and interpersonal relationships (i.e., client-provider interactions and client experience) as interrelated elements. Later frameworks redefined quality of family planning care with more a client-centered, right-based lens such as the 2014 World Health Organization recommendation for scaling up rights-based contraceptive program, the 2016 International Planned Parenthood Federation framework, and the 2018 Jain commentary [9–11].

The classic Donabedian quality framework defines what is needed to measure quality: structures, processes, and outcomes [12]. Structural quality is the setting of care; processes include the standards and elements of care delivery; and outcomes are the client-level health, behavior, knowledge, and satisfaction effects of the processes of care [12]. In general, we aim to measure process quality of care. Structural quality is a prerequisite but does not guarantee quality services and outcomes are difficult to measure and attribute to process/structural quality, likely due to the heterogenous measures, tools and definitions [7]. For instance, Weidert, et al. found positive associations between counseling on contraceptive methods and provider supervision with long-acting contraceptive use in Togo, and Chang et al. found significant but inconsistent associations of facility-level quality measures with method continuation across two sites in Pakistan and Uganda [13, 15].

To improve quality services, we need well-defined quality measures with a clear linkage to population-level impacts, and tools that reliably and accurately measure them. Many tools have been developed and implemented to measure process quality of care but no agreed-upon standardized tool or method has emerged [16, 17]. Process quality of care can be measured by assessor observations of client-provider interactions in clinical settings (also known as “direct observation”), interviewing clients after their family planning visits (“exit interviews”), provider interviews/quizzes or clinical vignettes on knowledge of quality care practices (“provider knowledge assessment”), medical record review, or simulated client assessments involving either trained staff or women recruited from the communities to act as “mystery” clients. These tools measure different elements: provider knowledge, provider practices and client perspectives of quality care but they all aim to capture the same construct of process quality.

Program implementers in LMICs could measure each of these elements on a routine basis to ensure quality services, but it may not be feasible to conduct a comprehensive quality of care assessment in some low-resource settings. Direct observation or simulated client methods are considered “gold standard” tools because they capture quality of care through direct observation of provider practices—either by an assessor during direct observation or covertly through a simulated client but these methods are not feasible to implement regularly. Direct observation may not be feasible due to the expense of field-based data collection and the time it takes to administer them and simulated clients may not be feasible due to the time and skill required to recruit and train the simulators. Other tools like client exit interviews or provider knowledge assessments may provide a proxy but there is little information on how these measures compare to the “gold standard” (validity). Information on validity or reliability (i.e., how well the tools measure the same element of care) will help implementers select the most appropriate tool for their program, the quality assessment aims, and the context.

We aim to synthesize the literature on validity and reliability of family planning quality of care measurement tools through a systematic review. We define validity as how well a tool measures provider actions during a family planning consultation compared to a “gold standard” assessment, usually an observed or simulated client-provider interaction—otherwise known as concurrent validity. Validity gives us information how well these tools proxy actual processes of care. Reliability describes how different tools measure the same quality indicator which gives us information on how to interpret the data they produce. The findings of this review can help refine and delineate best practices for quality of family planning care measurement.

## Methods

This study is a quantitative systematic review with no meta-analysis due to the heterogeneous methods, analysis and outcomes of the studies found during the review.

### Search strategy and selection criteria

We developed three search concepts: family planning, quality of care, and comparability of tool metrics with a filter for LMICs. We searched PubMed, Embase, Popline, Global Index Medicus and SCOPUS. The terms were pretested to see if the search would identify two relevant articles. Additional file 1: Appendix S1 lists all search terms used for the PubMed database. For the initial search, we extracted all relevant citations up through 17th October 2017, with follow-up searches done through 31st March 2019 and 16th March 2021. The Popline database was retired in September 2019, therefore only papers up until 31st March 2019 (the second search) were included. We searched ProQuest’s Dissertation and theses database for additional, relevant studies and identified authors currently working in family planning quality of care measurement and contacted them for any unpublished findings.

We included studies that quantitatively measured quality of care for family planning in LMICs (Box 1). The studies must have compared at least two tools (e.g. direct observation and client exit interviews) and reported a quantitative measurement of the comparison. We did not require that the authors report uncertainty measures such as standard errors or 95% confidence intervals. Google translate was used to screen French and Spanish language titles and abstracts, and a staff person with language fluency was recruited for full text review/data extraction of manuscripts not written in English. We restricted the studies to quality of care measurement tools for health providers (excluding school or peer-based assessments) and included all papers published through 16th March 2021.

Box 1: Eligibility criteria for inclusion of studies

- Family planning
- LMIC setting
- Process quality of care
- Quantitative data comparing the tools
- Sufficient detail on the tools and study protocol
- Measure quality of care from health providers
- Studies published up to 16^th^ March 2021

A team (E.H, S.K.S, P.S, M.J) conducted double-blind title/abstract screening and full text review and another researcher (D.M) independently resolved conflicts. Two researchers (P.K and M.J) conducted the data extraction and the corresponding author (E.H) verified and synthesized the findings. We summarized the search and selection process using a Preferred Reporting Items for Systematic Reviews and Meta-Analysis (PRISM) flowchart [18].

**Data extraction and analysis**

We were primarily interested in the comparison of similar quality measurements using different tools. We extracted pre-defined, descriptive elements for each study and the comparison measures organized by indicator and tool type. The comparison measure could be either a summative estimate such as sensitivity/specificity, likelihood ratios or positive/negative predictive values, or sufficient individual-level data for calculating a summative estimate. If a gold standard method was not identified in the study but two or more tools were quantitatively compared, we included kappa statistic, prevalence adjusted, bias adjusted kappa (PABAK), percent agreement or percentage points (pp) difference. We extracted comparison measures for all quality measurements presented in the studies and organized them into domains: counseling on method selection, method use, information on side effects and how to manage them, other general counseling, and privacy and respectful care. We then classified as low (< 0.4), medium (0.4–0.6) and high (> 0.6) agreement between the “gold standard” tool and the comparator tool based on the kappa, sensitivity, specificity, and predictive values. There is no consensus on what constitutes an acceptable level of agreement among survey tools, so we loosely followed the consensus for kappa statistic measuring interrater reliability [19, 20]. We used a similar ranking for percentage points (pp) difference between the tools (high comparability: < 15 pp, medium: 15–25 pp, low: > 25 pp difference).

We used the Critical Appraisal Skills Programme (CASP) diagnostic checklist as a guide for determining quality of the individual studies [21]. We categorized the studies into low, medium, and high risk of bias (Additional file 1: Appendix S2) and presented the study findings in tables with a narrative summary. We used Covidence software for title/abstract screening and full text review, and Office 365 Microsoft Excel for data abstraction and synthesis [22, 23]. This review was registered in Prospero (ID no. CRD42019136293) and the protocol is available online [24].

## Results

**Fig. 1:**
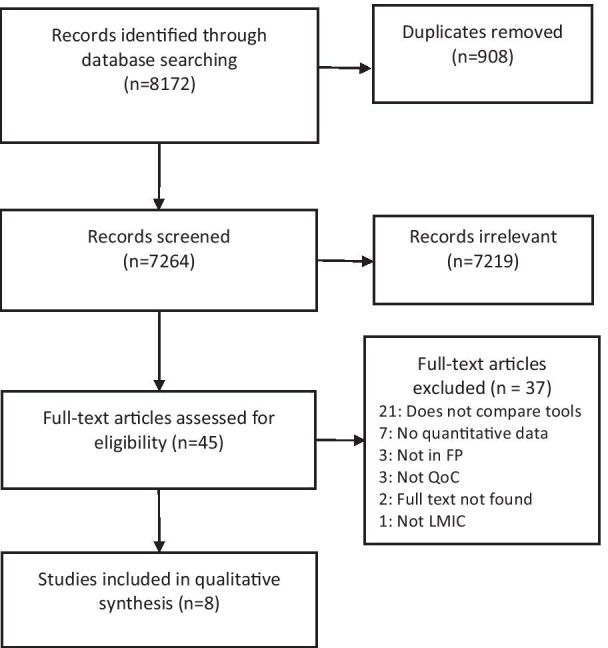
PRISM flow chart. Quality of care (QoC); family planning (FP); low-and-middle-income country (LMIC)

The search yielded 8172 articles with 908 duplicates (Fig. 1). The full-text review identified 37 articles for exclusion: 21 did not provide comparison on the measurements generated from two or more tools, seven did not include a quantitative comparison, seven studies did not take place in a LMIC setting or did not measure family planning quality of care. We were unable to find full text documents for two studies: one was meeting minutes from a 1995 meeting at University of North Carolina and the second was a 1998 report from the International Planned Parenthood Federation. We included eight studies in our final analysis [25–31].

The studies were a mix of single country and multi-country studies with data from Sub-Saharan Africa, South Asia and Central and South America (Table 1). They were published from 1998 to 2020, six were published in peer reviewed journals and two were not peer reviewed. Three were secondary data analysis of Service Provision Assessment (SPA) data (Choi 2018), Quick Investigation of Quality (QIQ) data (Bessinger 2001) or Situational analysis data (Ndhlovu 1998) and five were primary data collection studies. Direct observation (n = 5) and simulated client (n = 3) protocols were identified as the “gold-standard” or the tool in which other comparisons were evaluated against. Comparator tools included client exit interviews, provider interviews and medical record review. One study (Tumlinson 2014) compared direct observation to simulated clients as the “gold standard” since providers do not know they are being assessed during a simulated client encounter.

**Table 1:** Description of the seven studies included in the summative analysis

	Choi 2018	Tumlinson 2014	Bessinger 2001	Ratanajamit 2001	Hermida 1999	Ndhlov 1998	Tavrow 1997	Thongmixay 2020
Country	Haiti, Senegal, Malawi, Tanzania	Kenya	Ecuador, Uganda, Zimbabwe	Thailand	Guatemala	Kenya	Malawi	Lao PDR
QoC tools	SPA	Tools adapted from QIQ & MLE	QIQ	Cross-sectional study	Observation for 3 weeks	Situational Analysis	National QoC FP assessment	QIQ
Analysis type	Secondary data analysis	Primary data collection	Secondary data analysis	Primary data collection	Primary data collection	Secondary data analysis	Primary data collection	Primary data collection
“Gold standard” tool	Direct Observation	Simulated Client	Direct Observation	Simulated client	Direct observation	Direct Observation	Simulated client	Direct Observation
Comparator tool	Exit interview	Direct observation, Exit Interview, Provider Interview	Exit interview	Provider interview	Exit Interview, medical record review	Exit interview	Exit interview	Exit interview
Statistic	Sensitivity, specificity	% agreement, Sensitivity, Specificity, PPV/NPV, Likelihood Ratios	% agreement, PABAK	% agreement	Sensitivity, Specificity	Kappa	Percentage point difference	Approximate percentage point difference
Study participants	Facilities: 1868 Clients: 6429	Facilities: 19 Clients: 49	Facilities: 154 Clients: 1858	Facilities: 60 Clients: Female and male simulated clients were used	Facilities: 3 Clients: 67	Clients:240	Facilities: 39 Clients: 477	Facilities: 17 Clients interviewed: 393 Clients observed: 218
Provider type	Not mentioned	Registered or community nurses	Not mentioned	Private providers at drugstores	Physician, nurse, nursing auxiliary	Not mentioned	Not mentioned	Not mentioned
Methods evaluated	Pills, injectables, IUDs, implants, or female sterilization	Implants, injectables, IUDs oral contraceptives	IUDs, pills, barrier methods, and injectables	Oral contra-ceptives	Not mentioned	Not mentioned	Not mentioned	Not mentioned
Indicator	Counseling completeness for side effects	Choice of methods, information given to user, continuity mechanism, competency, interpersonal relationship, and continuity mechanism	Provider actions, counseling and interpersonal relationships	History taken and advice given	FP counseling	Counseling and methods mentioned	Counseling and encouraging client questions	Information discussed during consultations
Risk of bias	Low	Low	Low	Medium	Medium	High	Medium	Medium

Quality of care (QoC); Family planning (FP); Service Provision Assessment (SPA); Quick Investigation of Quality (QIQ); Measurement, Learning and Evaluation Project (MLE); Positive predictive value (PPV); Negative predictive value (NPV); Prevalence-adjusted and bias-adjusted kappa (PABAK); Intrauterine device (IUD); People's Democratic Republic (PDR)

A variety of test statistics were used to compare the tools and the sample size varied as well (range: 6429–49 clients), the secondary analysis studies pooling multi-country data tended to have larger samples compared to those conducting primary data analysis. Only three studies mentioned the provider type and four mentioned the type of contraceptive used in their quality assessment tools. All studies evaluated quality related to counseling, information given to the clients, interpersonal relationships, or respectful care. We identified one study as low risk of bias, six as medium risk and one as high risk of bias for the reported findings (Additional file 1: Appendix S2).

**Fig. 2:**
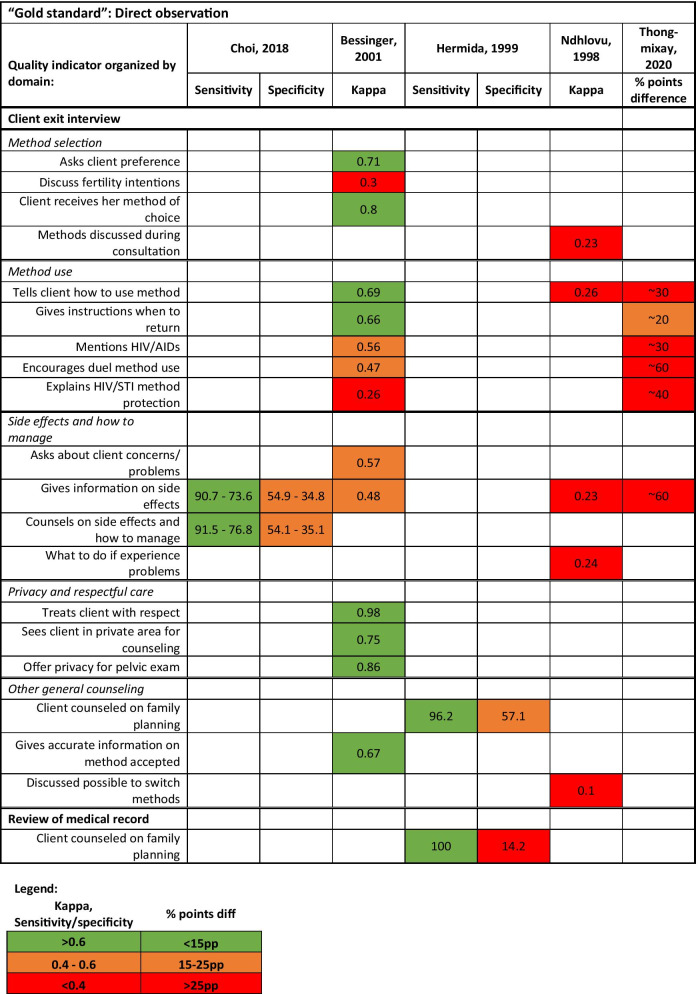
Quantitative measures comparing client exit interviews and medical record review with direct observation

Four of the eight studies reported validity measures using a gold standard: (Choi 2018 and Hermida 1999) using direct observation as gold standard and (Tumlinson 2014 and Ratanajamit 2001) using simulated client as gold standard) (Fig. 2) The four remaining studies reported kappa statistics (Bessinger 2001 and Ndhlovu 1998) or percentage point difference (Tavrow 1997 and Thongmixay 2020).

Most of the studies (n = 5) compared client exit interviews to direct observation (Fig. 2). Three of these studies reported comparability for counseling on method selection and use. The measurements varied from very poor agreement (kappa: 0.23) for “Methods discussed during consultation” to high agreement (kappa: 0.8) for “Client received her method of choice” (Fig. 2). Thongmixay (2020) reported percentage point (pp) differences from 20 to 60 pp depending on the indicator. Four studies investigated the comparability of side effects counseling as reported during the client interviews and as assessed during the direct observation. Again, these ranged widely based on the specific indicator. Choi, 2018 reported good sensitivity (90.7–73.6 country ranges) for “Gives information on side effects” while Ndhlovu, 1998 reported very low agreement (kappa: 0.23). Thongmixay (2020) found approximately a 60 pp discrepancy between client report and direct observation regarding whether the client was given information on the side effects.

Only Bessinger (2001) reported on the comparability of privacy and respectful care with high agreement (ranging from 0.75 to 0.98 kappa). We included a domain of “Other general counseling” for measurements that could not be easily grouped with other studies (Fig. 2). Hermida (1999) reported high sensitivity (96.2) and medium specificity (57.1) for whether the client received any counseling on family planning, Bessinger (2001) found high agreement on whether the client received accurate information on the method they received, and Ndhlovu (1998) found low agreement on whether the clients were told it was possible to switch methods. Hermida (1999) compared medical record review to direct observation and found high sensitivity (100%) and low specificity (14%) for whether the client received any family planning counseling (Fig. 2).

**Fig. 3:**
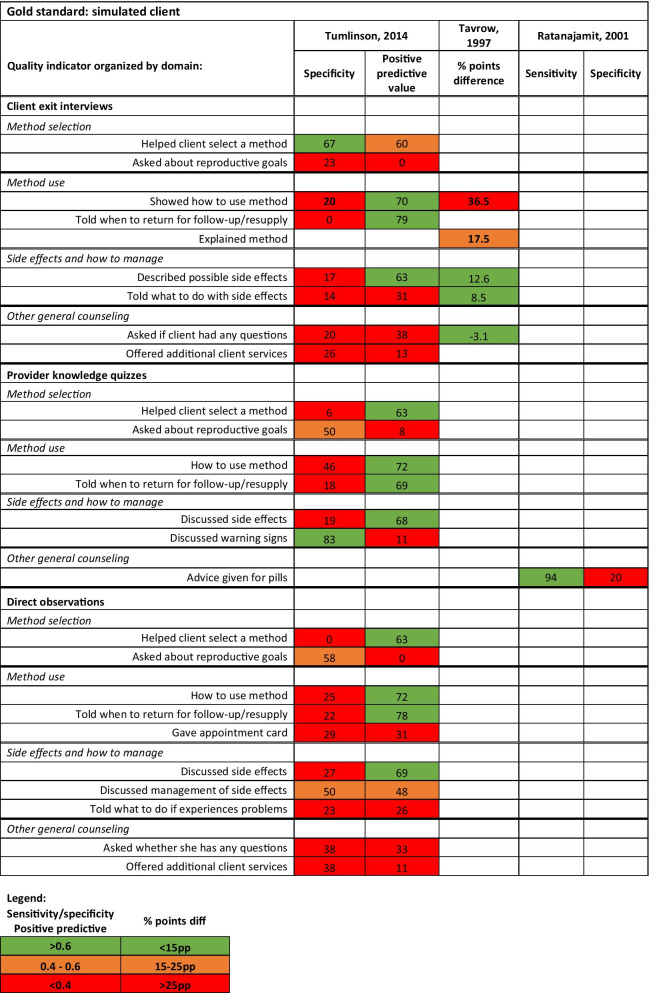
Quantitative measures comparing client exit interviews, provider interviews, and direct observation with simulated client

Most of the data using simulated client as the “gold standard” comes from one study (Fig. 3). Tumlinson (2014) compares client exit interviews, provider interviews and direct observations with simulated clients focusing on specificity as a validity measure. For instance, using direct observation as an example, did the health worker provide the same level of care during the direct observation when they knew they were being assessed as they did with the simulated clients when they (presumably) did not know it was a quality assessment. If specificity was lower, the health workers provided higher quality of care during the direct observation. Generally, they found low specificity except for two measurements: provider “helped client select a method” and “discussed warning signs”. For the first indicator, specificity was higher, 67% of the providers who did not help the client select a method for the simulated clients also did not do this for actual clients (as reported during the exit interviews). Similarly, for the second indicator, if the providers did not counsel the simulated client on danger signs, they did not mention this during the knowledge quizzes.

Tavrow, 1997 reported some degree of concurrence between simulated client and exit interviews as reflected in a low percentage point differences in the proportion of providers describing side effects (12.6 pp difference) and explaining how to manage them (8.5 pp difference) (Fig. 3). But a much higher proportion of simulated clients (83.6%) interviewed indicated that the provider showed the client how to use the method compared to what clients recounted during the exit interviews (47.1%) (36.5 pp difference). When comparing provider interviews quizzes and direct observation to simulated clients, Tumlinson, 2014 found poor specificity but higher positive predictive values depending on the indicator indicating provider have higher quality performance on quizzes and observational assessments compared to a simulated client, when they do not realize they are being assessed (Fig. 3). Similarly, Ratanajamit (2001), found high sensitivity (94%) and low specificity (20%) of the provider quizzes compared to simulated clients, indicative in the 2001 study that providers demonstrated higher level of knowledge than practiced.

## Discussion

Through our systematic review, we found only eight studies comparing measurements from family planning quality of care tools used in LMIC settings. These studies were heterogenous in their methods and in the quality measurements they defined and compared so there was insufficient data to estimate overall summary measures of validity or other comparison measures of the tools.

The problem with this heterogeneity is twofold. One, without better standardization of tools, indicators, and methods, it is difficult to understand program and policy impacts on quality care, especially for cross-country comparisons and time-trend analyses. Two, the heterogeneity means there is little data on the validity or reliability to guide tool selection for measuring process quality. For instance, it may not be feasible to conduct routine direct observations to monitor quality but other, less intensive protocols such as client interviews or clinical vignettes could be used as a proxy, depending on the program, the assessment aims, and the setting. More evidence is needed on the validity and reliability of these tools to guide tool selection for family planning program monitoring and evaluation.

Despite the difficulty comparing the tools, some patterns emerged within the limited information available. Overall, the specificity of measurements from client exit interviews was low, even when the sensitivity was high, clients were reporting health provider quality activities not recorded by the gold standard observation (n = 5 studies). Clients may have been reporting their general knowledge of family planning or experience with provider(s) they saw previously instead of during the visit being evaluated. Program implementers and evaluators should be cautious when using client exit interviews to measure provider quality actions. Other studies have found that women have difficulty accurately reporting on more technical aspects of quality care, particularly for delivery and newborn care. Two 2016 related studies in Kenya and Mexico found that women could not accurately report on some process quality delivery and newborn care measurements [32, 33]. A 2021 study in Bangladesh, Nepal and Tanzania, comparing direct observation and patient exit interview data found similarly low levels of validity [34]. This evidence suggests exit interviews may be more appropriate to measure client experience, perceptions, and general knowledge rather than technical quality. Although, exit interviews on experiential quality should be carefully interpreted since clients may respond more positively about their experience when interviewed exiting a facility compared to when interviewed at home (known as courtesy bias) [35, 36].

We have very limited information on the validity of medical record review and provider interviews. Hermida, 1999 found medical records to be an adequate tool for identifying providers that did not counsel on family planning (100% sensitivity for identifying performance failures) but it performed poorly for indicating whether the provider counseled the client since the providers would counsel the client but presumably forget to document this in the register (Fig. 2). Tumlinso (2014) and Ratanajamit (2001) found provider knowledge of quality activities to be higher than observed performance of those activities (low specificity). This discrepancy of higher provider knowledge related to lower performance is also known as the “know-do” gap and has been reported elsewhere in LMIC health systems [37–39]. Tumlison (2014) compared direct observation to simulated client and found little comparability of the methods where sample size was sufficient for comparison (Fig. 3). Summarizing from the eight studies, specificity for many quality measurements is low, evidence that provider may change their behavior due to the assessor observation, also known as reactivity bias [40].

Most of the quality measurements compared in these studies focus on counseling and interpersonal relationships. None reported more clinical, technical competencies like sterile technique or correct application of contraceptives, understandable since client exit interviews and simulated clients are non-clinicians and cannot accurately assess these clinical competencies. Even within each tool, the quality measurements across the studies are different. Some are subjective (“Helped client select a method” or “Treats client with respect”) where others are more objective (“Asks client preference” or “Mentions HIV/AIDS”). It is likely that the more subjective measurements vary by tool and have high inter-rater variability.

During the screening phase of the review, we found many studies using multiple tools for measuring family planning process quality that did not report any compatibility measures. And protocols used globally such as the SPA, QIQ, and other facility-based assessments include exit interview, provider interview and direct observations tools that could be compared to gauge reliability of the data. When performing a quality assessment, researchers should consider including some key quality measurements in multiple tools to test the reliability of the data, particularly studies that focus on client exit interviews for their quality of care measurements.

One limitation of this study is that we selected studies that specifically reported on validity or comparability of tools. It is possible many studies evaluated this but did not report it. We contacted several authors for unpublished data and searched grey literature databases to address this, but there still may be publication bias present.

## Conclusion

To measure family planning quality of care consistently and accurately in LMICs, a standardized suite of tools is needed along with an established method of combining them for a comprehensive picture of quality care. Heterogenous tools and metrics make it difficult to measure intervention or policy impacts on quality and to clearly describe the association of quality (e.g., counseling completeness) with outcomes (e.g., contraceptive continuation).

Family planning quality of care tools were crafted to measure different aspects of process quality: provider assessments measure knowledge, direct observations/simulated client protocols or medical record reviews measure provider practice, and exit interviews measure client knowledge, satisfaction, and experience. More research is needed on how well these tools proxy the actual processes of care provided to clients under everyday clinical conditions, especially since those interested in routine quality measures (annually or more frequently) are unlikely to have the time and resources to implement multiple tools. Selecting one or two methods is more feasible but there is little data on how the various tools compare and little guidance on which would be most appropriate for their context.

Although the number of studies in this review is small, there is emerging evidence of important differences in the same quality measurement produced by different tools. Though we have the most data on comparability of client exit interviews, they are a poor proxy of actual processes of care received and should be used primarily to measure client experience and knowledge. It is likely there are other important differences, but more studies of validity and comparability are needed.

Improving the reliability and accuracy of the methods used to measure quality of care will allow governments and program implementers to better monitor, understand, and improve quality and access of family planning services. As LMICs continue to scale-up quality-focused family planning services, accurate and timely measures of quality will inform and improve programs to reduce the unmet need for contraceptives meeting the goals laid out in the SGDs, the promises of universal health care coverage, and access to quality care as a basic human right [11].

## Supplementary Information

**Additional file 1: Appendix S1. **Search terms by three concepts and filter for PubMed database.** Appendix S2. **Risk of bias assessment.

## Data Availability

All data generated or analyzed during this study are included in this published article and its Additional file 1: Appendices.

## Abbreviations

CASP: Critical Appraisal Skills Programme
FP: Family planning
IUD: Intrauterine device
LMIC: Low-and-middle income countries
MLE: Measurement, Learning and Evaluation Project
NPV: Negative predictive value
PABAK: Prevalence-adjusted and bias-adjusted kappa
PP: Percentage points
PPV: Positive predictive value
PRISM: Preferred Reporting Items for Systematic Reviews and Meta-Analysis
QIQ: Quick Investigation of Quality
QoC: Quality of care
RADAR: Real Accountability: Data Analysis for Results
SPA: Service Provision Assessment
