# Supplementary material for: Comparability of family planning quality of care measurement tools in low-and-middle income country settings: a systematic review
Source: Reprod Health. 2021 Oct 30;18:215. doi: 10.1186/s12978-021-01261-1 (PMC8557007; doi:10.1186/s12978-021-01261-1)
Supplement: Supplementary file 1 — Additional file 1: Appendix S1. Search terms by three concepts and filter for PubMed database. Appendix S2. Risk of bias assessment. [file 12978_2021_1261_MOESM1_ESM.docx]

**Additional file 1: Appendices**

**Appendix S1: Search terms by three concepts and filter for PubMed database.**

**Concept 1: Family planning**

"family planning services"[MeSH Terms] OR “Family planning services”[Text Word] OR “Family planning service”[Text Word] OR “Family planning programs”[Text Word] OR “Family planning programmes”[Text Word] OR “Family planning programme”[Text Word] OR “Family planning program”[Text Word] OR “Family Planning Methods”[Text word] OR “Family Planning Method”[Text word] OR “Contraception”[MeSH terms] OR “Contraception”[Text Word] OR “Contraceptions”[Text Word] OR “Contraceptive”[Text Word] OR “Birth Control”[Text word] OR “Fertility Control”[Text word] OR "Reproductive Health"[Mesh] OR "Reproductive Health"[text word] OR "Reproductive Health Services"[Mesh] OR “Delayed Childbearing”[text word] OR "birth intervals"[MeSH Terms] OR “birth intervals"[text word] OR “birth interval"[text word] OR “birth spacing”[Text Word] OR “birth spacings”[Text Word]

**Concept 2: Quality of care**

“Quality Improvement” [MeSH Terms] OR “Quality Indicators, Health Care” [MeSH Terms] OR “Quality of Health Care” [MeSH Terms] OR “Quality of Health Care” [Text Word] OR “Quality Improvement” [Text Word] OR “Quality Indicators, Health Care” [Text Word] OR “Quality of Care” [Text Word] OR “Quality of services” [Text Word] OR “Service provision” [Text Word] OR “Quality of HealthCare” [Text Word]

**Concept 3: Comparability**

“Outcome and Process Assessment (Health Care)” [MeSH Terms] OR "Validation Studies" [Publication Type] OR validity[tiab] OR validation[tiab] OR validated [tiab] OR measurement[tiab] OR tool[tiab] OR tools[tiab] OR assess [tiab] OR assessment[tiab] OR assessing[tiab]

**Filter: LMICs**

(“emerging country”[all fields] OR “emerging countries”[all fields] OR “emerging nation”[all fields] OR “emerging nations”[all fields] OR “emerging population”[all fields] OR “emerging populations”[all fields] OR "developing country"[tiab] OR "developing countries"[tiab] OR "developing nation"[tiab] OR "developing nations"[tiab] OR "developing population"[tiab] OR "developing populations"[tiab] OR "developing world"[tiab] OR "less developed country"[tiab] OR "less developed countries"[tiab] OR "less developed nation"[tiab] OR "less developed nations"[tiab] OR "less developed population"[tiab] OR "less developed populations"[tiab] OR "less developed world"[tiab] OR "lesser developed country"[tiab] OR "lesser developed countries"[tiab] OR "lesser developed nation"[tiab] OR "lesser developed nations"[tiab] OR "lesser developed population"[tiab] OR "lesser developed populations"[tiab] OR "lesser developed world"[tiab] OR "under developed country"[tiab] OR "under developed countries"[tiab] OR "under developed nation"[tiab] OR "under developed nations"[tiab] OR "under developed population"[tiab] OR "under developed populations"[tiab] OR "under developed world"[tiab] OR "underdeveloped country"[tiab] OR "underdeveloped countries"[tiab] OR "underdeveloped nation"[tiab] OR "underdeveloped nations"[tiab] OR "underdeveloped population"[tiab] OR "underdeveloped populations"[tiab] OR "underdeveloped world"[tiab] OR "middle income country"[tiab] OR "middle income countries"[tiab] OR "middle income nation"[tiab] OR "middle income nations"[tiab] OR "middle income population"[tiab] OR "middle income populations"[tiab] OR "low income country"[tiab] OR "low income countries"[tiab] OR "low income nation"[tiab] OR "low income nations"[tiab] OR "low income population"[tiab] OR "low income populations"[tiab] OR "lower income country"[tiab] OR "lower income countries"[tiab] OR "lower income nation"[tiab] OR "lower income nations"[tiab] OR "lower income population"[tiab] OR "lower income populations"[tiab] OR "underserved country"[tiab] OR "underserved countries"[tiab] OR "underserved nation"[tiab] OR "underserved nations"[tiab] OR "underserved population"[tiab] OR "underserved populations"[tiab] OR "underserved world"[tiab] OR "under served country"[tiab] OR "under served countries"[tiab] OR "under served nation"[tiab] OR "under served nations"[tiab] OR "under served population"[tiab] OR "under served populations"[tiab] OR "under served world"[tiab] OR "deprived country"[tiab] OR "deprived countries"[tiab] OR "deprived nation"[tiab] OR "deprived nations"[tiab] OR "deprived population"[tiab] OR "deprived populations"[tiab] OR "deprived world"[tiab] OR "poor country"[tiab] OR "poor countries"[tiab] OR "poor nation"[tiab] OR "poor nations"[tiab] OR "poor population"[tiab] OR "poor populations"[tiab] OR "poor world"[tiab] OR "poorer country"[tiab] OR "poorer countries"[tiab] OR "poorer nation"[tiab] OR "poorer nations"[tiab] OR "poorer population"[tiab] OR "poorer populations"[tiab] OR "poorer world"[tiab] OR "developing economy"[tiab] OR "developing economies"[tiab] OR "less developed economy"[tiab] OR "less developed economies"[tiab] OR "lesser developed economy"[tiab] OR "lesser developed economies"[tiab] OR "under developed economy"[tiab] OR "under developed economies"[tiab] OR "underdeveloped economy"[tiab] OR "underdeveloped economies"[tiab] OR "middle income economy"[tiab] OR "middle income economies"[tiab] OR "low income economy"[tiab] OR "low income economies"[tiab] OR "lower income economy"[tiab] OR "lower income economies"[tiab] OR "low gdp"[tiab] OR "low gnp"[tiab] OR "low gross domestic"[tiab] OR "low gross national"[tiab] OR "lower gdp"[tiab] OR "lower gnp"[tiab] OR "lower gross domestic"[tiab] OR "lower gross national"[tiab] OR lmic[tiab] OR lmics[tiab] OR "third world"[tiab] OR "lami country"[tiab] OR "lami countries"[tiab] OR "transitional country"[tiab] OR "transitional countries"[tiab] OR Africa[tiab] OR Asia[tiab] OR Caribbean[tiab] OR West Indies[tiab] OR South America[tiab] OR Latin America[tiab] OR Central America[tiab] OR "Atlantic Islands"[tiab] OR "Commonwealth of Independent States"[tiab] OR "Pacific Islands"[tiab] OR "Indian Ocean Islands"[tiab] OR "Eastern Europe"[tiab] OR Afghanistan[tiab] OR Albania[tiab] OR Algeria[tiab] OR Angola[tiab] OR Armenia[tiab] OR Armenian[tiab] OR Azerbaijan[tiab] OR Bangladesh[tiab] OR Benin[tiab] OR Byelarus[tiab] OR Byelorussian[tiab] OR Belarus[tiab] OR Belorussian[tiab] OR Belorussia[tiab] OR Belize[tiab] OR Bhutan[tiab] OR Bolivia[tiab] OR Bosnia[tiab] OR Herzegovina[tiab] OR Hercegovina[tiab] OR Botswana[tiab] OR Brasil[tiab] OR Brazil[tiab] OR Bulgaria[tiab] OR Burkina Faso[tiab] OR Burkina Fasso[tiab] OR Upper Volta[tiab] OR Burundi[tiab] OR Urundi[tiab] OR Cambodia[tiab] OR Khmer Republic[tiab] OR Kampuchea[tiab] OR Cameroon[tiab] OR Cameroons[tiab] OR Cameron[tiab] OR Cape Verde[tiab] OR Central African Republic[tiab] OR Chad[tiab] OR China[tiab] OR Colombia[tiab] OR Comoros[tiab] OR Comoro Islands[tiab] OR Comores[tiab] OR Mayotte[tiab] OR Congo[tiab] OR Zaire[tiab] OR Costa Rica[tiab] OR Cote d'Ivoire[tiab] OR Ivory Coast[tiab] OR Cuba[tiab] OR Czechoslovakia[tiab] OR Slovakia[tiab] OR Djibouti[tiab] OR French Somaliland[tiab] OR Dominica[tiab] OR Dominican Republic[tiab] OR East Timor[tiab] OR East Timur[tiab] OR Timor Leste[tiab] OR Ecuador[tiab] OR Egypt[tiab] OR El Salvador[tiab] OR Eritrea[tiab] OR Ethiopia[tiab] OR Fiji[tiab] OR Gabon[tiab] OR Gabonese Republic[tiab] OR Gambia[tiab] OR Gaza[tiab] OR Georgia Republic[tiab] OR Georgian Republic[tiab] OR Ghana[tiab] OR Gold Coast[tiab] OR Grenada[tiab] OR Guatemala[tiab] OR Guinea[tiab] OR Guiana[tiab] OR Guyana[tiab] OR Haiti[tiab] OR Honduras[tiab] OR India[tiab] OR Maldives[tiab] OR Indonesia[tiab] OR Iran[tiab] OR Iraq[tiab] OR Jamaica[tiab] OR Jordan[tiab] OR Kazakhstan[tiab] OR Kazakh[tiab] OR Kenya[tiab] OR Kiribati[tiab] OR Korea[tiab] OR Kosovo[tiab] OR Kyrgyzstan[tiab] OR Kirghizia[tiab] OR Kyrgyz Republic[tiab] OR Kirghiz[tiab] OR Kirgizstan[tiab] OR "Lao PDR"[tiab] OR Laos[tiab] OR Lebanon[tiab] OR Lesotho[tiab] OR Basutoland[tiab] OR Liberia[tiab] OR Libya[tiab] OR Macedonia[tiab] OR Madagascar[tiab] OR Malagasy Republic[tiab] OR Malaysia[tiab] OR Malaya[tiab] OR Malay[tiab] OR Sabah[tiab] OR Sarawak[tiab] OR Malawi[tiab] OR Nyasaland[tiab] OR Mali[tiab] OR Marshall Islands[tiab] OR Mauritania[tiab] OR Mauritius[tiab] OR Agalega Islands[tiab] OR "Melanesia"[tiab] OR Mexico[tiab] OR Micronesia[tiab] OR Middle East[tiab] OR Moldova[tiab] OR Moldovia[tiab] OR Moldovian[tiab] OR Mongolia[tiab] OR Montenegro[tiab] OR Morocco[tiab] OR Ifni[tiab] OR Mozambique[tiab] OR Myanmar[tiab] OR Myanma[tiab] OR Burma[tiab] OR Namibia[tiab] OR Nepal[tiab] OR Nicaragua[tiab] OR Niger[tiab] OR Nigeria[tiab] OR Muscat[tiab] OR Pakistan[tiab] OR Palau[tiab] OR Palestine[tiab] OR Panama[tiab] OR Paraguay[tiab] OR Peru[tiab] OR Philippines[tiab] OR Philipines[tiab] OR Phillipines[tiab] OR Phillippines[tiab] OR Romania[tiab] OR Rumania[tiab] OR Roumania[tiab] OR Rwanda[tiab] OR Ruanda[tiab] OR Saint Kitts[tiab] OR St Kitts[tiab] OR Nevis[tiab] OR Saint Lucia[tiab] OR St Lucia[tiab] OR Saint Vincent[tiab] OR St Vincent[tiab] OR Grenadines[tiab] OR Samoa[tiab] OR Samoan Islands[tiab] OR Navigator Island[tiab] OR Navigator Islands[tiab] OR Sao Tome[tiab] OR Senegal[tiab] OR Serbia[tiab] OR Montenegro[tiab] OR Sierra Leone[tiab] OR Sri Lanka[tiab] OR Ceylon[tiab] OR Solomon Islands[tiab] OR Somalia[tiab] OR Sudan[tiab] OR Suriname[tiab] OR Surinam[tiab] OR Swaziland[tiab] OR Syria[tiab] OR Syrian[tiab] OR Tajikistan[tiab] OR Tadzhikistan[tiab] OR Tadjikistan[tiab] OR Tadzhik[tiab] OR Tanzania[tiab] OR Thailand[tiab] OR Togo[tiab] OR Togolese Republic[tiab] OR Tonga[tiab] OR Tunisia[tiab] OR Turkey[tiab] OR Turkmenistan[tiab] OR Turkmen[tiab] OR Tuvalu[tiab] OR Uganda[tiab] OR Ukraine[tiab] OR Uzbekistan[tiab] OR Uzbek OR Vanuatu[tiab] OR New Hebrides[tiab] OR Vietnam[tiab] OR Viet Nam[tiab] OR West Bank[tiab] OR Yemen[tiab] OR Yugoslavia[tiab] OR Zambia[tiab] OR Zimbabwe[tiab] OR Rhodesia[tiab] OR Developing Countries[Mesh] OR Africa[Mesh:NoExp] OR Africa, Northern[Mesh:NoExp] OR Africa South of the Sahara[Mesh:NoExp] OR Africa, Central[Mesh:NoExp] OR Africa, Eastern[Mesh:NoExp] OR Africa, Southern[Mesh:NoExp] OR Africa, Western[Mesh:NoExp] OR Asia[Mesh:NoExp] OR Asia, Central[Mesh:NoExp] OR Asia, Southeastern[Mesh:NoExp] OR Asia, Western[Mesh:NoExp] OR Caribbean Region[Mesh:NoExp] OR West Indies[Mesh:NoExp] OR South America[Mesh:NoExp] OR Latin America[Mesh:NoExp] OR Central America[Mesh:NoExp] OR "Atlantic Islands"[Mesh:NoExp] OR "Commonwealth of Independent States"[Mesh:NoExp] OR "Pacific Islands"[Mesh:NoExp] OR "Indian Ocean Islands"[Mesh:NoExp] OR "Europe, Eastern"[Mesh:NoExp] OR Afghanistan[Mesh] OR Albania[Mesh] OR Algeria[Mesh] OR American Samoa[Mesh] OR Angola[Mesh] OR Armenia[Mesh] OR Azerbaijan[Mesh] OR "Baltic States"[Mesh] OR Bangladesh[Mesh] OR Benin[Mesh] OR "Republic of Belarus"[Mesh] OR Belize[Mesh] OR Bhutan[Mesh] OR Bolivia[Mesh] OR Bosnia-Herzegovina[Mesh] OR Botswana[Mesh] OR Brazil[Mesh] OR Bulgaria[Mesh] OR Burkina Faso[Mesh] OR Burundi[Mesh] OR Cambodia[Mesh] OR Cameroon[Mesh] OR Cape Verde[Mesh] OR Central African Republic[Mesh] OR Chad[Mesh]] OR China[Mesh] OR Colombia[Mesh] OR Comoros[Mesh] OR Congo[Mesh] OR Costa Rica[Mesh] OR Cote d'Ivoire[Mesh] OR Cuba[Mesh] OR Czechoslovakia[Mesh] OR Slovakia[Mesh] OR Djibouti[Mesh] OR "Democratic Republic of the Congo"[Mesh] OR "Democratic People's Republic of Korea"[Mesh] OR Dominica[Mesh] OR Dominican Republic[Mesh] OR East Timor[Mesh] OR Ecuador[Mesh] OR Egypt[Mesh] OR El Salvador[Mesh] OR Eritrea[Mesh] OR Ethiopia[Mesh] OR Fiji[Mesh] OR "French Guiana"[Mesh] OR Gabon[Mesh] OR Gambia[Mesh] OR "Georgia (Republic)"[Mesh] OR Ghana[Mesh] OR Grenada[Mesh] OR Guatemala[Mesh] OR Guinea[Mesh] OR Guinea-Bissau[Mesh] OR Guyana[Mesh] OR Haiti[Mesh] OR Honduras[Mesh] OR "Independent State of Samoa"[Mesh] OR India[Mesh] OR Indonesia[Mesh] OR Iran[Mesh] OR Iraq[Mesh] OR Jamaica[Mesh] OR Jordan[Mesh] OR Kazakhstan[Mesh] OR Kenya[Mesh] OR Korea[Mesh] OR Kyrgyzstan[Mesh] OR Laos[Mesh] OR Lebanon[Mesh] OR Lesotho[Mesh] OR Liberia[Mesh] OR Libya[Mesh] OR "Macedonia (Republic)"[Mesh] OR Madagascar[Mesh] OR Malawi[Mesh] OR Malaysia[Mesh] OR Mali[Mesh] OR Mauritania[Mesh] OR Mauritius[Mesh] OR "Melanesia"[Mesh] OR Mexico[Mesh] OR Micronesia[Mesh] OR Middle East[Mesh:NoExp] OR Moldova[Mesh] OR Mongolia[Mesh] OR Montenegro[Mesh] OR Morocco[Mesh] OR Mozambique[Mesh] OR Myanmar[Mesh] OR Namibia[Mesh] OR Nepal[Mesh] OR Nicaragua[Mesh] OR Niger[Mesh] OR Nigeria[Mesh] OR Pakistan[Mesh] OR Palau[Mesh] OR Panama[Mesh] OR Papua New Guinea[Mesh] OR Paraguay[Mesh] OR Peru[Mesh] OR Philippines[Mesh] OR "Republic of Korea"[Mesh] OR Romania[Mesh] OR Rwanda[Mesh] OR Saint Lucia[Mesh] OR "Saint Vincent and the Grenadines"[Mesh] OR Samoa[Mesh] OR Senegal[Mesh] OR Serbia[Mesh] OR Montenegro[Mesh] OR Sierra Leone[Mesh] OR Sri Lanka[Mesh] OR Somalia[Mesh] OR South Africa[Mesh] OR Sudan[Mesh] OR Suriname[Mesh] OR Swaziland[Mesh] OR Syria[Mesh] OR Tajikistan[Mesh] OR Tanzania[Mesh] OR Thailand[Mesh] OR Togo[Mesh] OR Tonga[Mesh] OR Tunisia[Mesh] OR Turkey[Mesh] OR Turkmenistan[Mesh] OR Uganda[Mesh] OR Ukraine[Mesh] OR Uzbekistan[Mesh] OR Vanuatu[Mesh] OR Vietnam[Mesh] OR Yemen[Mesh] OR Yugoslavia[Mesh] OR Zambia[Mesh] OR Zimbabwe[Mesh] OR “Southern African Development Community”[all fields] OR “East African Community"[all fields] OR “West African Health Organisation"[all fields] OR “Sub Saharan Africa "[all fields] OR “SubSaharan Africa "[all fields])

**Appendix S2: Risk of bias assessment**

| **CASP question (adapted):** | **Choi, 2018** | **Tumlinson, 2014** | **Bessinger, 2001** | **Ratanajamit, 2001** | **Hermida, 1999** | **Ndhlovu, 1998** | **Tavrow, 1997** | **Thongmixay, 2020** |
| --- | --- | --- | --- | --- | --- | --- | --- | --- |
| Was there a clear question for the study to address? | Yes | Yes | Yes | Yes | Yes | No | Yes | Yes |
| Were the tools administered on the same client or clinic for comparability? | Yes, DO and CEI on some client (SPA) | Yes, same providers | Yes, DO and CEI on some client (QIQ) | Yes, same clinic measured 1 day apart | Yes, same patients used | Yes, DO and CEI on some client (Situational Analysis) | Yes, same clinic with SC and CEI interviews | Yes, DO and CEI on some client (QIQ) |
| Could the results of one tool have been influenced by the results of another tool? | Yes - CEI given immediately after visit | Less likely because SC was used | Yes - CEI given immediately after visit | Less likely because SC was used | Unclear - little detail is given on interview and medical record reviews | Unclear | Less likely because SC was used | Yes - CEI given immediately after visit |
| Were the tool methods described in sufficient detail? | No but SPA methods are reported elsewhere | Yes | Yes | Yes | Unclear - little detail is given on interview and medical record reviews | No | Yes | Yes |
| How sure are we about the results? | Good - shows CI, SPA is random sample | Good - shows CI; convenience sample | No uncertainly estimates for kappa; convenience sample | No CIs, random sample | No uncertainly estimates for kappa; convenience sample | Okay - shows p value for kappa. | Good p values and random sample | No CIs, random sample |
| Are the results generalizable to other LMICs? | Yes - mutli-country in LMIC | Yes - Kenya only but LMIC | Yes - multi country - combination of public/private sectors | Perhaps - private clinics in Thailand only | Perhaps - although provider type was mostly physician; different from many LMICs | Yes - Kenya only by LMIC | Yes | Yes – Lao PDR only but LMIC |
| Were all quality indicators considered? | No - covered only a few on counseling | Yes - covered many quality indicators in analysis | Yes - covered many quality indicators in analysis | No | No - only covered one on counseling | No - only covered a few on counseling | Yes - covered many quality indicators in analysis | Yes - covered many quality indicators in analysis |
| Risk of bias and rationale | Low | Medium: small sample size | Medium: CI but sample size was high, convenience sample | Medium - not same setting, comparability data was ancillary analysis | Medium: small sample sizes, no CI and FP was not main outcome | High - not peer reviewed, comparability data was ancillary analysis | Medium - not peer reviewed | Medium – peer reviewed, adequate sample size, no CIs shown. |

Acronyms: Service Provision Assessment (SPA); simulated client (SC); client exit interview (CEI); 95% confidence intervals (CI); low-and-middle income country (LMIC); direct observation (DO); family planning (FP); Quick Investigation of Quality (QIQ); People's Democratic Republic (PDR)

**Description of bias assessment:**

We identified one study (Choi, 2018) as low risk of bias because the sample size was high, generalizability was high (multiple LMICs included in analysis) and the uncertainty estimates around the comparison statistics were shown (appendix 2). Six studies were identified as medium risk of bias. Tumlinson, 2014 has small sample size with non-randomly selected clinics but they showed uncertainity estimates for the statistics and use of simulated client is a less biased “gold standard” compared to direct observation due to reactivity bias. Bessinger, 2001 was considered medium risk of bias due to lack of uncertainity estimates (although sample size was high) and the clinics were not randomly selected. However, generalizability is high since it was a multi-country LMIC study with public and private sector facilities. We considered Ratanajamit, 2001 as medium risk of bias. They randomized clinic selection but did not include uncertainity estimates, and generalizability for our research question is low because the setting is private-for-profit drugstores in Thailand, classified as a lower middle-income country in 2001. We considered Hermina, 1999 as medium risk of bias because of smaller sample sizes, lack of uncertainity estimates and family planning was an ancillary analysis. The dissertation chapter by Tavrow, 1997 had a decent sample size, included uncertainity estimates, a randomized sample, and used simulated client protocol as the gold standard but it was not peer-reviewed so we designated it as medium bias risk. Finally, Thongmixay, 2020 was designated medium risk of bias because there were no uncertainly estimates reported and only bar graphs are shown for the measurement comparison allowing only an approximate percentage point difference to be extracted. However, the sample size was larger (n=218 direct observations) and the setting (public sector facilities in Lao PDR) is generalizable to other LMICs. One study (Ndhlovu, 1998) was deemed high risk of bias because the comparability analysis was ancillary, and the study was not peer-reviewed.
